# Granulosa secreted factors improve the developmental competence of cumulus oocyte complexes from small antral follicles in sheep

**DOI:** 10.1371/journal.pone.0229043

**Published:** 2020-03-17

**Authors:** Shiva Rouhollahi Varnosfaderani, Mehdi Hajian, Farnoosh Jafarpour, Faezeh Ghazvini Zadegan, Mohammad Hossein Nasr-Esfahani

**Affiliations:** Department of Reproductive Biotechnology, Reproductive Biomedicine Research Center, Royan Institute for Biotechnology, ACECR, Isfahan, Iran; Friedrich-Loeffler-Institute, GERMANY

## Abstract

Oocyte *in vitro* maturation can be improved by mimicking the intra-follicular environment. Oocyte, cumulus cells, granulosa cells, and circulating factors act as meiotic regulators in follicles and maintain oocyte in the meiotic phase until oocyte becomes competent and ready to be ovulated. In a randomized experimental design, an ovine model was used to optimize the standard *in vitro* maturation media by Granulosa secreted factors. **At first, the** development capacity of oocyte derived from medium (>4 to 6 mm) and small (2 to ≤4 mm) size follicles was determined. Differential gene expression of granulosa secreted factors and their receptors were compared between the cumulus cells of the two groups. Then, the best time and concentration for arresting oocytes at the germinal vesicle stage by natriuretic peptide type C (CNP) were determined by nuclear staining in both groups. Oocyte quality was further confirmed by calcein uptake and gene expression. The developmental competence of cumulus oocyte complexes derived from small size follicles that were cultured in the presence of CNP in combination with amphiregulin (AREG) and prostaglandin E2 (PGE2) for 24 h was determined. Finally, embryo quality was specified by assessing expressions of *NANOG*, *SOX2*, *CDX2*, *OCT4*, and *TET1*. The cumulus oocyte complexes derived from small size follicles had a lower capacity to form blastocyst in comparison with cumulus oocyte complexes derived from medium size follicles. Prostaglandin E receptor 2 and prostaglandin-endoperoxide synthase 2 had significantly lower expression in cumulus cells derived from small size follicles in comparison with cumulus cells derived from medium size follicles. Natriuretic peptide type C increased the percentage of cumulus oocyte complexes arresting at the germinal vesicle stage in both oocytes derived from medium and small follicles. Gap junction communication was also improved in the presence of natriuretic peptide type C. In oocytes derived from small size follicles; best blastocyst rates were achieved by sequential exposure of cumulus oocyte complexes in [TCM+CNP (6 h), then cultured in TCM+AREG+PGE2 (18h)] and [TCM+CNP (6 h), then cultured in conventional IVM supplements+AREG+PGE2 (18h)]. Increased *SOX2* expression was observed in [TCM+CNP (6 h), then cultured in TCM+AREG+PGE2 (18h)], while decreased OCT4 expression was observed in [TCM+CNP (6 h), then cultured in conventional IVM supplements+AREG+PGE2 (18h)]. It seems that the natriuretic peptide type C modulates meiotic progression, and oocyte development is probably mediated by amphiregulin and prostaglandin E2. These results may provide an alternative IVM method to optimize *in vitro* embryo production in sheep and subsequently for humans.

## Introduction

Perhaps the greatest challenge of assisted reproductive techniques is *in vitro* maturation. Edwards reported the first IVM in humans, and Cha showed the first live birth after IVM in a woman with premature ovarian failure (POF). Since then, a great result has been achieved by capacitation pre-maturation-IVM (CAPA-IVM). Despite this recent progress in CAPA-IVM, this method has not replaced the conventional IVM procedure in humans and other species [1–3].

Improved development of *in vitro* matured oocytes can only be achieved by expanding our knowledge regarding the complex dialog between the oocyte and its surrounding somatic cells within follicle [4,5]. Nevertheless, this has proven to be challenging because: 1) dealing with a heterogeneous population of oocytes harvested from follicles of different size (2–6 mm in sheep and goat, 2–8 mm in bovine and < 12mm in human) is a formidable issue; 2) cumulus oocyte complexes (COCs) derived from small antral follicles possess less competence in response to regulatory and specific ligands because of an immature signaling capacity [6–10]; and 3) Isolation of COCs from their natural follicular environment results in spontaneous meiotic progression and, thus, asynchronization of nuclear and cytoplasmic maturation [11].

Recent microarray analyses between developmentally competent and incompetent COCs identified differential expression of quality marker genes in human and bovine [12–15]. Based on these studies, critical deficiencies in IVM may be related to a lack of granulosa cell-COC communication. The well-known factors secreted from granulosa cells (GCs) during *in vivo* maturation process are natriuretic peptides (NPs), epidermal growth factor (EGF)-like factors, and prostaglandins (PGs) that regulated extracellular cellular matrix, metabolism and immune system [12,16–19].

Members of the NP peptides include atrial natriuretic peptide (ANP), brain natriuretic peptide (BNP), and c-type natriuretic peptide (CNP). They are secreted by granulosa cells [20]. CNP is considered as the main NP and binds to natriuretic peptide receptor 2 (NPR2) on CCs, inducing the production of cyclic guanosine monophosphate (cGMP). Cyclic GMP enters oocyte via GJC and regulates levels of cyclic adenosine monophosphate (cAMP) by suppressing the hydrolyzing activity of oocyte-specific phosphodiesterases 3A (PDE3A). Increased cAMP level maintains meiotic arrest of immature oocytes within follicles [14, 21–23]. Besides, it has been stated that estradiol can mediate the expression of *NPR2* on CCs [22].

EGF-like factors, AREG, epiregulin (EREG), and betacellulin (BTC) act on EGF receptor (EGFR) and activate the extracellular signal-regulated protein kinases 1 and 2 (ERK1/2), protein kinase C (PKC) pathways and other signaling pathways in granulosa and cumulus cells (CCs), which make the COCs competent to respond to Luteinizing hormone (LH) surge [17, 24]. One of the target genes of the ERK1/2 pathway is prostaglandin synthase 2 (*PTGS2*), which leads to the production of PGE2 through a positive feedback loop between AREG and PGE2. Prostaglandin E2 (PGE2), an arachidonic acid-derived lipid mediator, is an autocrine/paracrine factor that mediates gonadotrophin (Gn) stimulation of cumulus expansion and oocyte maturation [16, 25–28].

Culture of COCs in medium containing granulosa secreted factors has been suggested to increase the proximity of *in vitro* culture to *in vivo* condition and improve the efficiency of *in vitro* oocyte maturation, especially in COCs derived from small follicles (2 to ≤ 4mm) with lower developmental competence compared to follicles of ≥ 4 to 6mm or of greater size [29, 30]. Our goal is to select ovine as a model for human IVM in future studies.

To achieve this aim, we initially exposed COCs from small follicles to CNP for 6 h and then to TCM 199 as a base of routine culture medium in the presence of AREG and/or PGE2, and the results of each group were compared with their corresponding group.

## Materials and methods

All procedures were approved by the Institutional Review Board of the Royan Institute (the ethical guidelines established by the Institutional Ethical Committee of the Royan Institute). The majority of chemicals and media were obtained from Sigma Chemical Co. (St. Louis, MO, USA) and Gibco (Grand Island, NY, USA), respectively. Other chemicals used for specific experiments from other companies are cited in the text as required.

### *In vitro* maturation (IVM)

**Fig 1:**
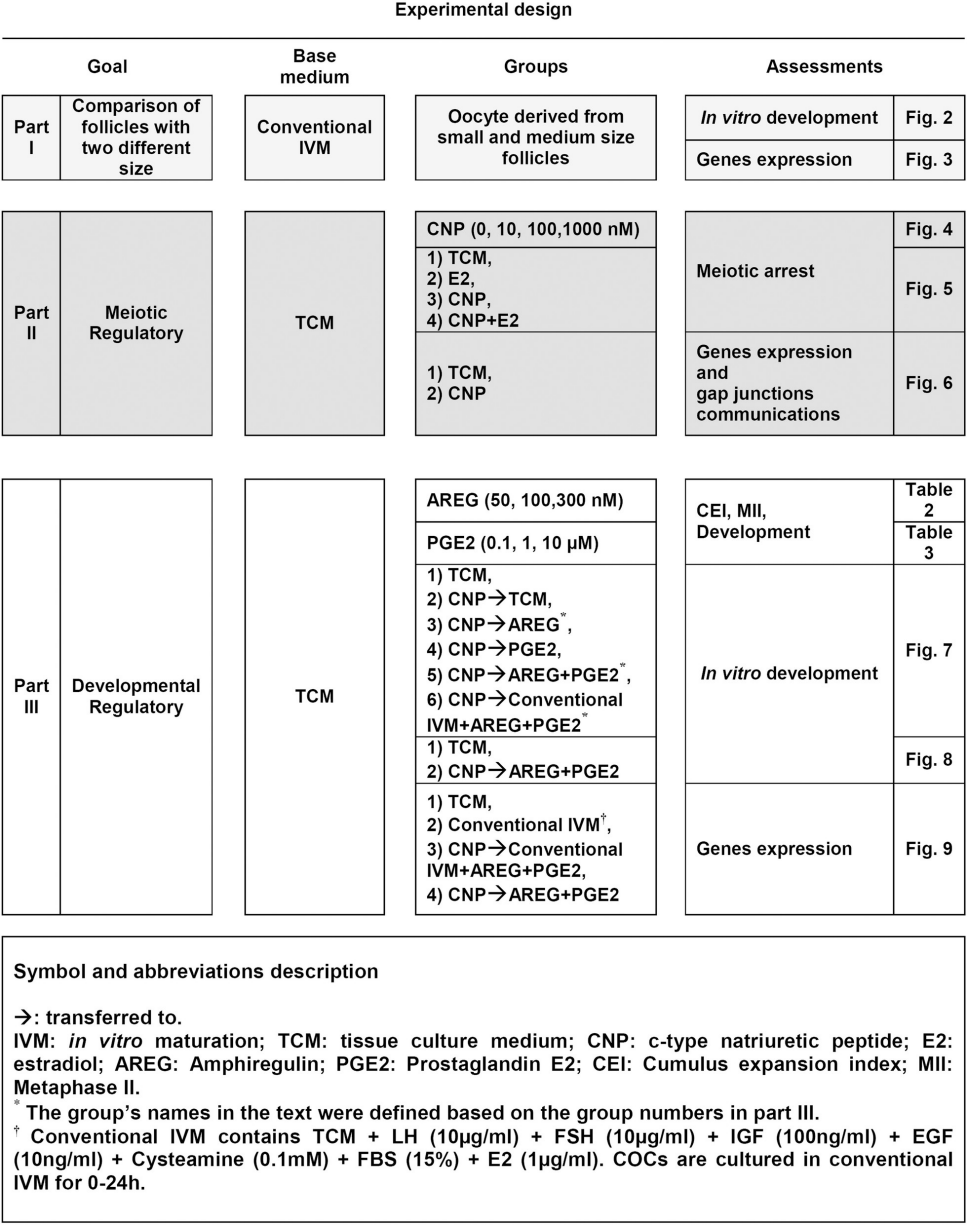
Experimental design.

Ovine ovaries from a local abattoir were transported to the laboratory at 6:00 pm in saline (15°C–20°C) and stored for an additional 12 h at 15°C since the lab working hours start in the morning [31, 32]. COCs were isolated from medium (≥ 4 to 6 mm) and small (2 to ≤ 4 mm) size follicles with the aid of 21-G needles. COCs with more than three layers of cumulus cells and homogenous cytoplasm were washed with the HEPES tissue culture medium 199 (HTCM199) + 1 mg/mL PVA+ 4 mg/mL BSA. Finally, 50 COCs were cultured in 400 μl containing 1 mg/mL PVA+ 8 mg/mL BSA in each group, according to experimental design (Fig 1) in 4 well dishes without mineral oil for 24 h at 38.5°C and 5% CO_2_ in the air [33, 34].

### Nuclear status

COCs were treated with 300 IU/mL hyaluronidase and vortexes to remove CCs. Denuded oocytes (DOs) were subsequently fixed for 20 minutes in 4% paraformaldehyde. To visualize chromatin, DOs were stained with Hoechst 33342 (10 μg/mL) for 5 minutes. After mounting, images of stained oocytes were captured and assessed by high-resolution digital camera (DP-72 Olympus, Japan) using DP2-BSW software [35]. The percentage of oocytes at the GV stage in each group was determined.

### Cumulus expansion index (CEI)

After 24 h IVM, CEI was scored on a 0 to 4 scale, as described by Vanderhyden et al., 1999: score 0, no expansion; score 1, no CC expansion but cells appear as spherical; score 2, only the outermost layers of CCs have expanded; score 3, all cell layers have expanded except the corona radiata; and score 4, expansion has occurred in all cell layers including the corona radiate [36].

### *In vitro* fertilization (IVF)

Domestic sheep breed, Rouge de l'ouest, was used for *in vitro* fertilization. Motile sperms were separated using a swim-up preparation; 100 μl of fresh sperm (from a ram of proven fertility) was kept under Tyrode’s albumin lactate pyruvate medium in 5% CO_2_, 38.5°C, and humidified air for up to 45 minutes to allow motile sperm to swim up. Subsequently, insemination was carried out by adding 5×10^3^ sperm/ matured COCs in fertilization medium (NaCl 114 mM, KCl 3.15 mM, NaH_2_PO_4_ 0.39 mM, Na-lactate 13.3 mM, CaCl_2_ 2 mM, MgCl_2_ 0.5 mM, Na-pyruvate 0.2 mM, Penicillin 50 IU/ml, Streptomycin 50 μg/ml, NaHCO_3_ 25 mM, Heparin 10 μg/ml, 6 mg/ml BSA) for 20 h at 38.5°C under 5% CO_2_ in humidified air overlaid with light mineral oil. On the next day, to remove the cumulus cells, the presumptive zygotes were vortexed in HTCM199 + 1mg/ml PVA+ 4mg/ml BSA for 3 minutes [37]. Then, they were cultured for 8 days in BO-IVC (Brackett-Oliphant *in vitro* culture) medium (Bioscience, UK) at 39°C, 6% CO_2_, 5% O_2_ in humidified air under mineral oil. Day 0 was defined as the day of insemination. Therefore, cleavage and blastocyst rates (over cleavage) were determined on days 3 and 7 post embryo culture.

### Relative gene expression

In each group, at the desired time, CCs were collected from COCs after vortexing for 4 minutes. Then, CCs were separated from oocytes, and CCs were stored in RLT buffer at -70°C until RNA extraction. RNA was extracted from CCs with the aid of an RNeasyMini Kit (Qiagen) followed by DNase I (Fermenas; EN0521) treatment. Total RNA (1000 ng) was reverse-transcribed using a Takara cDNA Synthesis kit (Takara; #RR07A) according to the manufacturer’s instructions. The primers were designed using Beacon and Oligo7 softwares. Efficiency correction for each primer was performed by serial dilution of positive control cDNA as a template. Please note that we are comparing the level of expression within different periods; we have no control group. Therefore, the transcripts abundance of 6 genes (*AREG*, *EGFR*, *NPPC*, *NPR2*, *PTGER2*, *PTGS2*, *GJA4*, and *GJA1*) (Table 1) was normalized to B-actin as reference gene using 2^^-(delta CT)^ rather than using 2^^-(delta delta CT)^.

**Table 1:** Primer sequences.

Gene symbol	Forward primer (5^´^-3^´^)	Reverse primer (5^´^-3^´^)	Annealing temp. (°C)
*OCT4*	GGAAAGGTGTTCAGCCA	ATTCTCGTTGTTGTCAGC	57
*NANOG*	CCTCTCAACATACAGCC	TCTTATTGGACTCATTACC	51
*SOX2*	GAGAACAATGACACACCAA	TGCTGAAATGAGGAGGAG	57
*CDX2*	CCCCAAGTGAAAACCAG	TGAGAGCCCCAGTGTG	56
*TET1*	CGGAAGAAAGAAGGTCGTC	GAATAACACCAAATGAGCGG	57
*AREG*	ATACTGCTGGATTAGATG	CTGTGGTTCATTATCATAC	49
*EGFR*	ACAAGACAATAAGCCACTT	CACCCAAAGGAGAGAAAG	50
*NPPC*	CCAATCTCAAGGACGAC	TTGGACAAACCCTTCTT	53
*NPR2*	AACTCCACTCTCAACTCTG	CTCTGAATTGCCGAACTG	56
*PTGS2*	CTTCCAGCCGCAGTAG	GGCATCTATGTCTCCGTA	58
*PTGER2*	CATCCTGAGACCTCCTGTTC	CTACCACTTCTTAACTACCATCCT	58
*GJA4*	TCCTTCCTAATGACCAGAG	GTAAGTTGTCTCCGAATCC	53
*GJA1*	GTGTCGTTGGTGTCTCTTG	CAGTGGTAGTGTGGTAAGGA	61
*β-actin*	CCATCGGCAATGAGCGGT	CGTGTTGGCGTAGAGGTC	58

Total RNA of five blastocysts in each group that was stored in RLT buffer at -70°C was extracted with the aid of Micro- RNeasy kit (Qiagen, Canada). For reverse transcription, 14 μl of total RNA (1μg) was used in a final volume of 20 μl reaction that contained 1 μl of random hexamer, 2 μl RT buffer (10x), 1 μl of RNase inhibitor (40 IU), 1 μl of reverse transcriptase (Takara; #RR07A), and 1 μl DEPC water. Reverse transcription was carried out at 37°C for 15 minutes, followed by 85°C for 5 seconds. Moreover, real-time PCR was implemented using 1 μl of cDNA (50 ng), 5 μl of the SYBR Green qPCR Master Mix (2x) (Fermentas, Germany) and 1 μl of forward and reverse primers (5 pM) adjusted to a total volume of 10 μl using nuclease-free water. Real-time PCR program was 1) 95°C 4 min, 2) 94°C 10 s, 3) Ta 30 s, and 4) 72°C 30 s, for 40 cycles. The transcripts abundance of 5 genes (nanog homeobox (*NANOG)*, SRY-Box transcription factor 2 (*SOX2)*, caudal type homeobox 2 (*CDX2)*, octamer-binding transcription factor 4 (*OCT4)* and ten-eleven translocation methylcytosine dioxygenase 1 *(TET1)*) (Table 1) were normalized to beta-actin as reference gene, and 2^^-(delta delta CT)^ was presented.

### Statistical analysis

**Fig 2:**
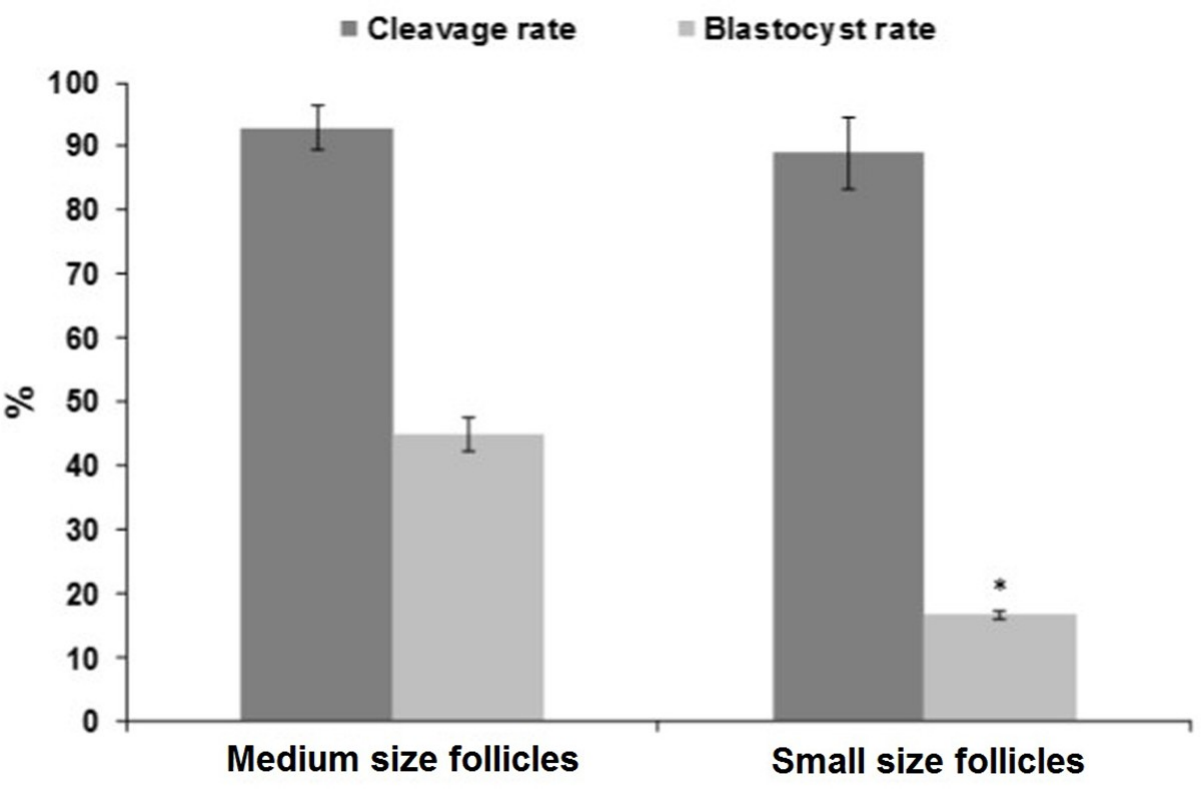
Developmental competency of *in vitro* conventional maturation of COCs derived from medium (>4 to 6 mm) compared to small (2 to ≤4 mm) size follicles. 5 replicates and minimum number of oocytes in each replicate were 50. Culture medium was conventional maturation medium containing TCM + LH (10μg/ml) + FSH (10μg/ml) + IGF (100ng/ml) + EGF (10ng/ml) + Cys (0.1mM) + FBS (15%) + E2 (1μg/ml). The asterisk represents a significant difference (P<0.05) within columns with the same color.

**Fig 3:**
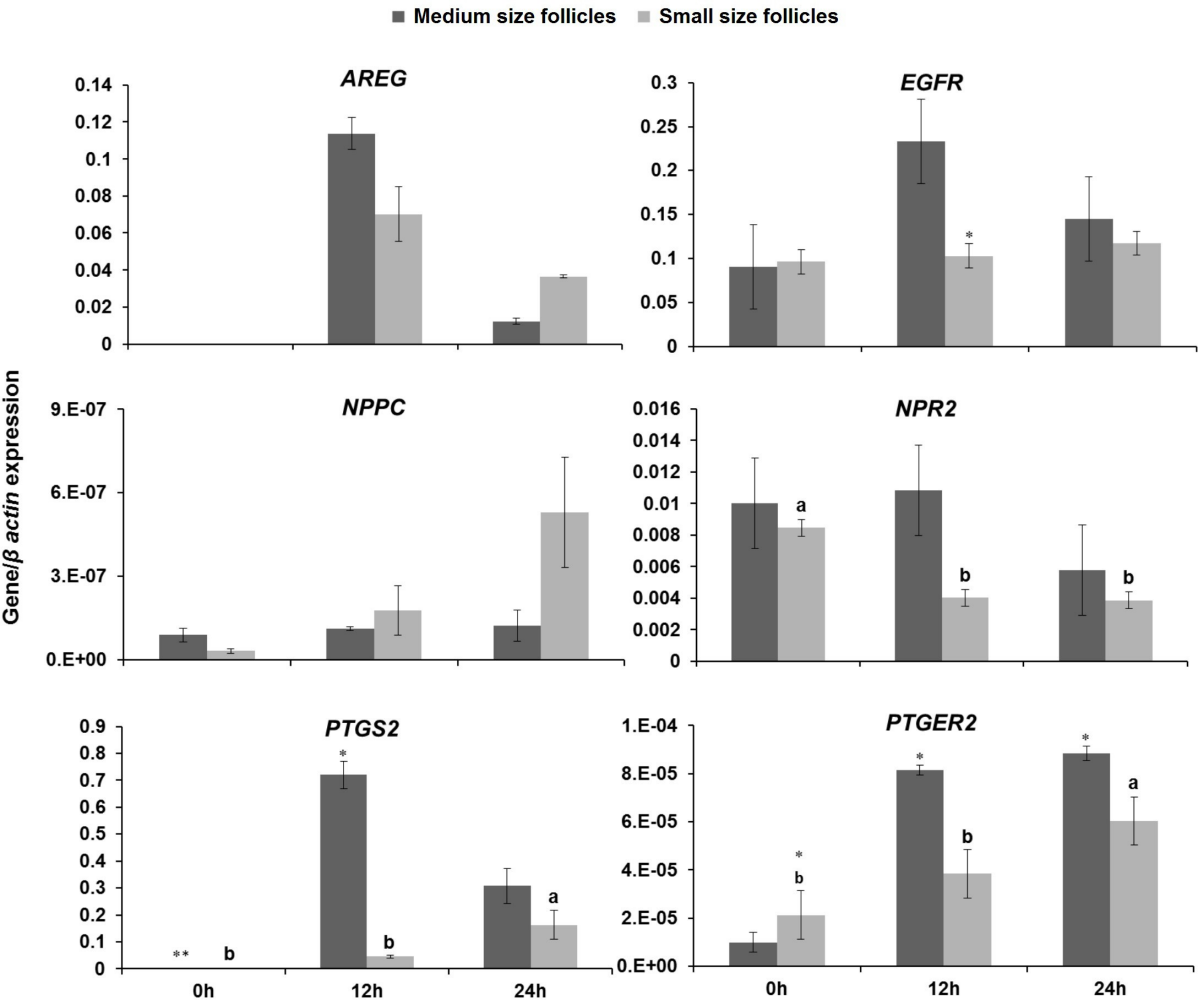
Gene expression of *AREG*, *EGFR*, *NPPC*, *NPR2*, *PGE2*, and *PTGS2* between cumulus cells derived from medium (>4 to 6 mm) and small (2 to ≤4 mm) size follicles at 0, 12 and 24 h post- *in vitro* maturation in a conventional medium. 3 replicates and minimum number of cumulus cells in each replicate were 2×10^5^. Different superscripts demonstrate significant differences (P<0.05) within columns with the same color. The asterisks represent significant differences between the two groups at the same time of maturation. 2^^-(delta CT)^ was presented for gene expression. *AREG*: amphiregulin, *NPPC*: natriuretic peptide precursor C, *PTGS2*: prostaglandin-endoperoxide synthase 2, *EGFR*: epidermal growth factor receptor, *NPR2*: natriuretic peptide receptor B, *PGE2*: prostaglandin E synthase 2.

**Fig 4:**
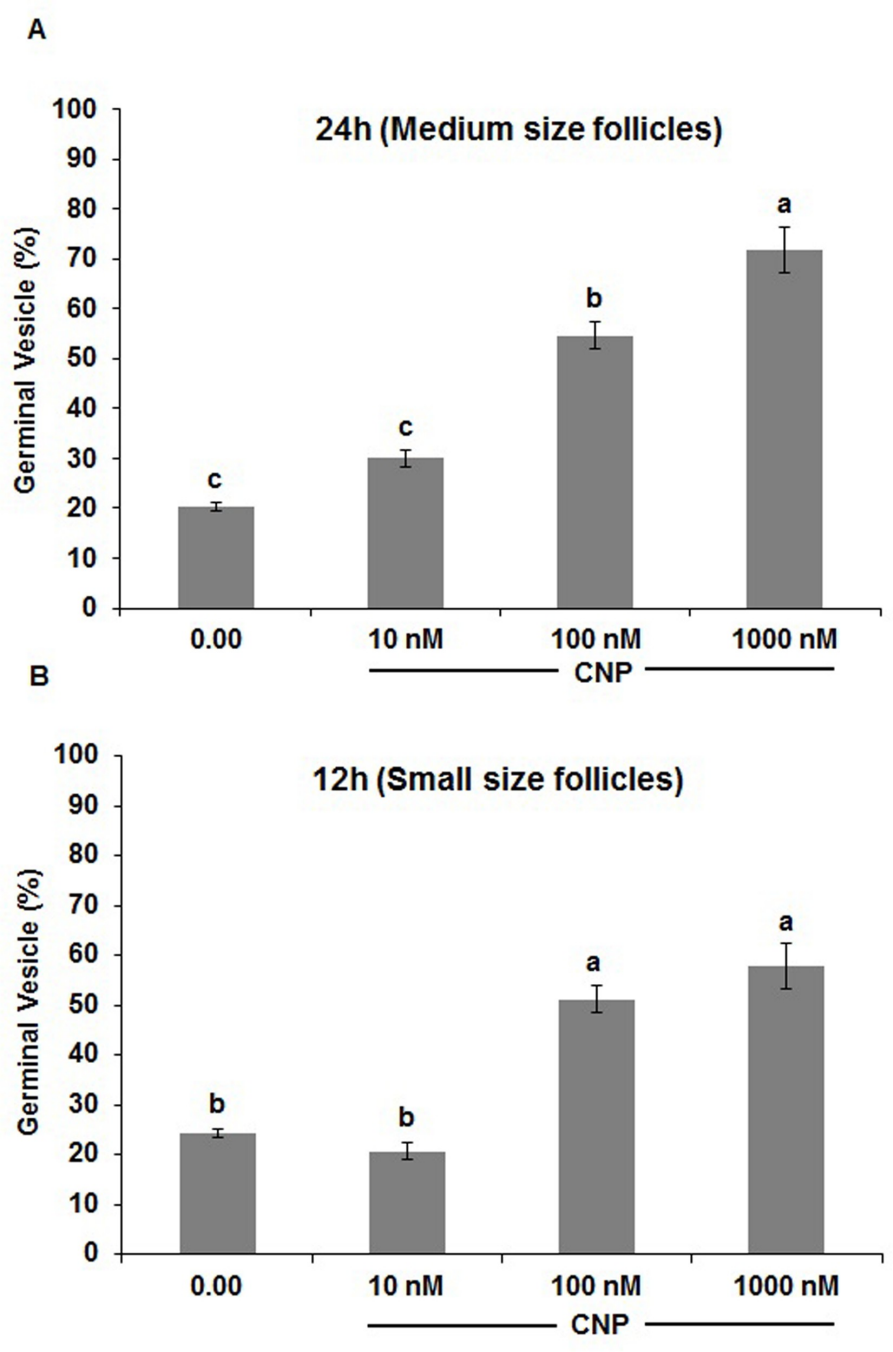
Determination of optimal concentration of CNP between oocytes derived from medium (>4 to 6 mm) and small (2 to ≤4 mm) size follicles. 3 replicates and the minimum number of oocytes in each replicate were 30. Different superscripts demonstrate significant differences (P<0.05). CNP: natriuretic peptide type C.

**Fig 5:**
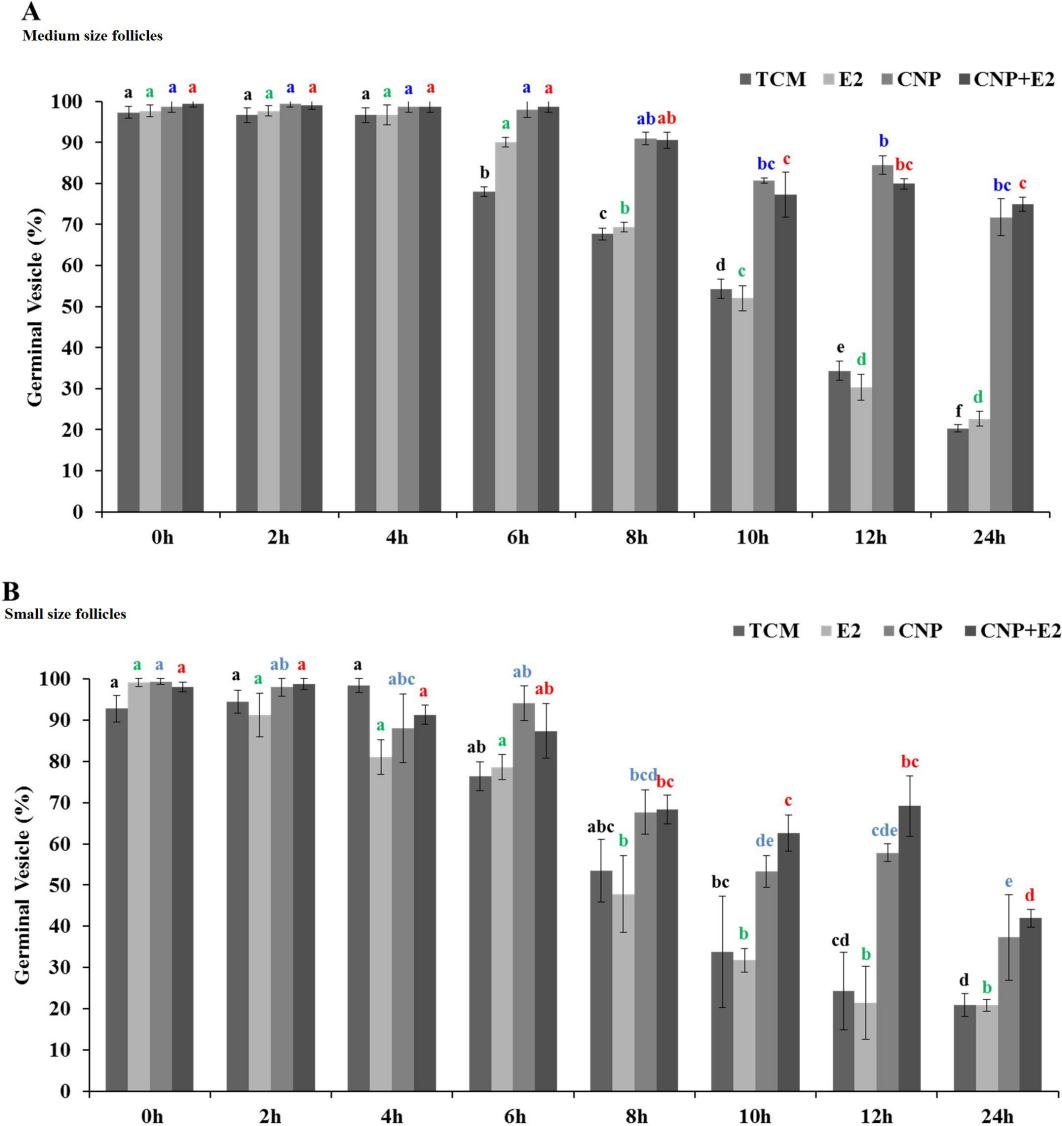
Percentage of oocytes at the GV stage during 24 h IVM in four experimental groups (TCM, E2, CNP, CNP+E2). **A**: medium size follicles (>4 to 6 mm) **B**: small size follicles (2 to ≤4 mm). 3 replicates and the minimum number of oocytes in each replicate were 20. Different superscripts demonstrate significant differences (P<0.05) within columns with the same color. TCM: tissue culture medium, E2: estradiol, CNP: natriuretic peptide type C.

**Fig 6:**
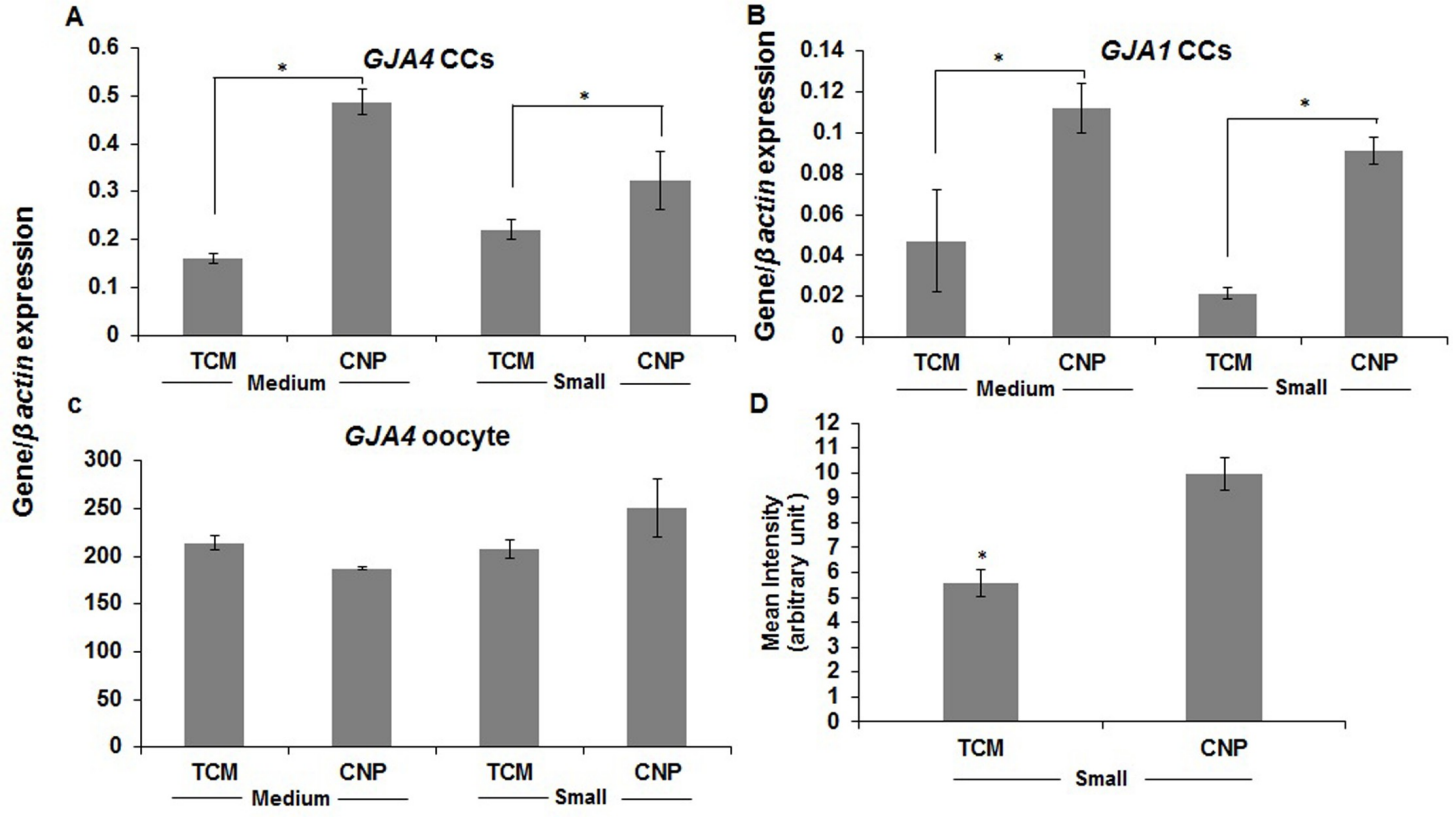
**A**, **B**, **C**: Gene expression of *GJA4* and *GJA1* between TCM and CNP groups in medium (>4 to 6 mm) and small (2 to ≤4 mm) size follicles. 3 replicates and the minimum number of oocytes and cumulus cells in each replicate were 30 and 2×10^5^ cells, respectively. **D**: Calcein uptake through gap junctions between TCM and CNP groups in COCs derived from small size follicles (2 to ≤4 mm). 3 replicates and the minimum number of oocytes in each replicate were 10. The asterisks represent a significant difference. GJA: gap junction protein alpha, TCM: tissue culture medium, CNP: natriuretic peptide type C.

**Fig 7:**
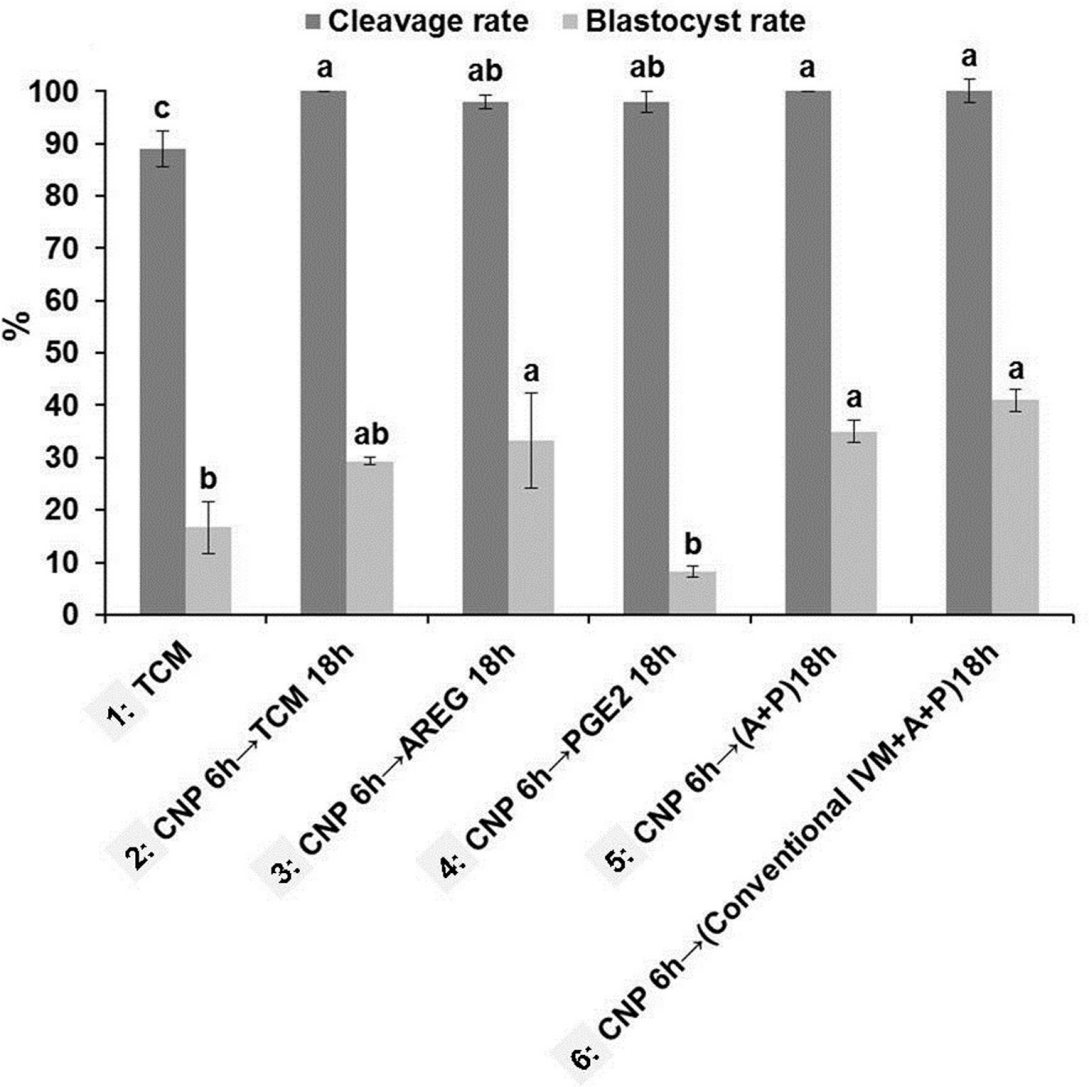
Developmental competence of COCs harvested from small size follicles (2 to ≤4 mm) in sheep sequentially exposed to CNP for 6 h, then cultured in TCM+PGE2 and/or AREG for 18 h. Different superscripts demonstrated significant differences (P<0.05) within columns with the same color. 5 replicates and the minimum number of oocytes in each replicate were 50. TCM: tissue culture medium, CNP: natriuretic peptide type C, AREG: amphiregulin, PGE2: prostaglandin E2.

**Fig 8:**
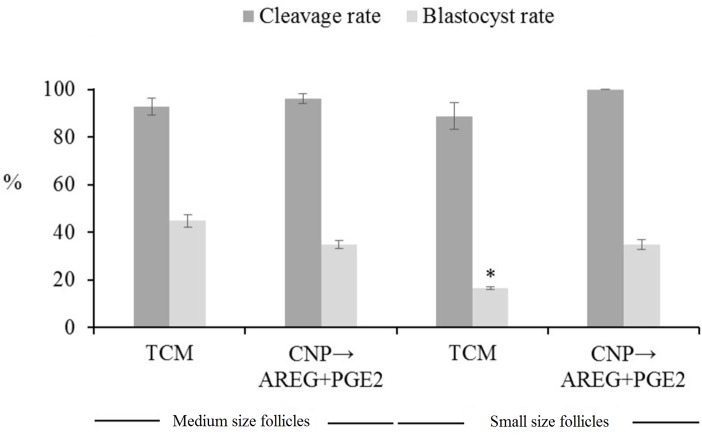
*In vitro* development of COCs derived from medium (>4 to 6 mm) compared to small (2 to ≤4 mm) size follicles that cultured in TCM (control) or TCM+NP then cultured in TCM+AREG+PGE2 (treatment). 5 replicates and the minimum number of oocytes in each replicate were 50. The asterisks represent the significant difference for blastocyst rate in small size follicles. TCM: tissue culture medium, CNP: natriuretic peptide type C, AREG: amphiregulin, PGE2: prostaglandin E2.

**Fig 9:**
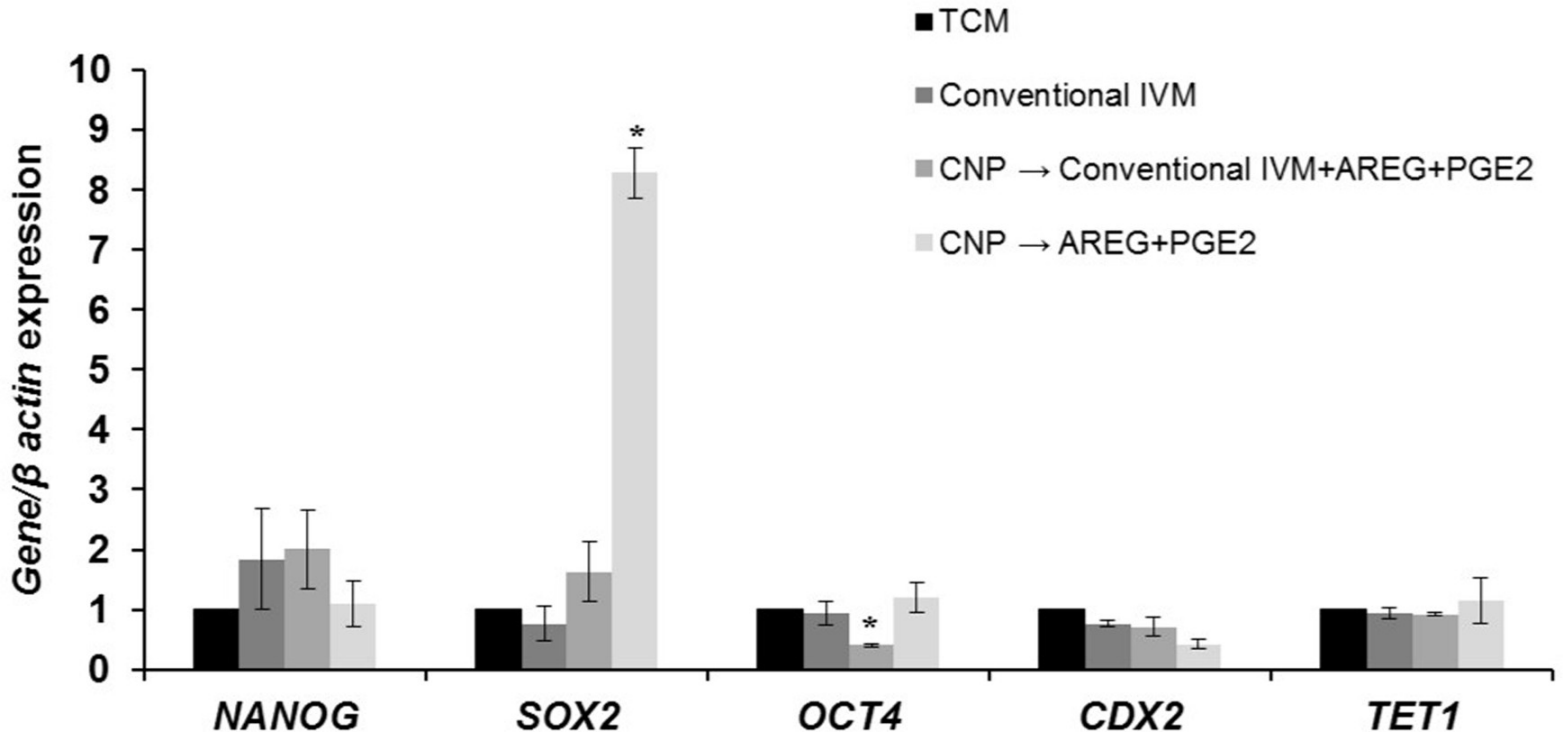
Assessment of relative expression of pluripotency and epigenetic markers in blastocysts derived from TCM, conventional IVM (24 h), [TCM+CNP (6 h), then cultured in TCM+AREG+PGE2 (18 h)] and [TCM+CNP (6 h), then cultured in conventional IVM supplements + AREG + PGE2 (18 h)] groups. 3 replicates and the minimum number of blastocysts in each replicate were 5. 2^^-(delta delta CT)^ was presented. The asterisks represent significant differences within columns with the same color. TCM: tissue culture medium, CNP: natriuretic peptide type C, AREG: amphiregulin, PGE2: prostaglandin E2.

Data percentages were modeled to a normal distribution by ArcSin transformation. Cleavage, blastocysts rates, and relative gene expression results among more than three groups were examined using a one-way ANOVA followed by Tukey’s post hoc tests (Figs 3–5, Fig 7 and Fig 9). The t-test was used for cleavage, blastocysts rates, and relative gene expression data between two groups (Fig 2, Fig 6 and Fig 8). The differences were considered significant at P<0.05. All results were presented as means ± standard error of the mean (SEM).

## Results

### Comparison of developmental competence and gene expression between COCs derived from medium (>4 to 6 mm) and small (2 to ≤4 mm) size follicles

The COCs derived from small size follicles had a lower capacity to form blastocyst in comparison with COCs derived from medium size follicles [16.6 ± 5.0 vs. 42.9 ± 3.5; p<0.05; Fig 2; see the experimental design (Fig 1, part I)].

No significant difference was found for *AREG* gene expression between CCs derived from two groups at 3 time points. *EGFR* gene expression in CCs derived from medium size follicles at 12 h post-maturation was significantly higher than small size follicles (p<0.05). No significant difference was found for *NPPC* gene expression between CCs derived from two groups at 3 time points. *NPR2 gene* expression significantly declined during the first 12 h of maturation in CCs derived from small size follicles (p<0.05). Expressions of *PTGER2* and *PTGS2 genes* between two groups at 3 time points were lower in CCs derived from small size follicles, except for *PTGER2* that had higher expression in small compared with medium size follicles at 0h post-maturation (p<0.05, Fig 3).

### Effects of CNP on meiotic arrest, relative gene expression, and gap junction’s communications

As the concentration of CNP increased (10, 100, 1000 nM), the percentage of oocytes derived from medium follicles remaining at the GV stage increased (P<0.05). In another group, there was no significant difference between 100 and 1000 nM of CNP (P>0.05, Fig 4). According to these results, concentrations of 1000 nM and 100 nM for CNP were selected for both medium and small size follicles, respectively [Fig 4, see the experimental design (Fig 1, part II)].

Meiotic progression of oocytes derived from medium follicles occurred around 6 h after the onset of IVM in TCM. The percentage of oocytes derived from medium size follicles at the GV stage after 24 h IVM was 20.3 ± 0.8 in TCM (Fig 5A). In this group, in the presence of CNP (1000 nM), the percentage of oocytes arrested at the GV stage after 24 h IVM was 71.7 ± 4.4 [Fig 5A, see the experimental design (Fig 1, part II)].

Meiotic progression of oocytes derived from small size follicles occurred around 8 h after the onset of IVM in TCM. The percentage of oocytes derived from small size follicles at the GV stage after 24 h IVM was 20.9 ± 2 in TCM (Fig 5B). In this group, in the presence of CNP (100 nM), the percentage of oocytes arrested at the GV stage after 24 h IVM was 37.3 ± 10.3 [Fig 5B, see the experimental design (Fig 1, part II)]. Our results also showed E2 independently or in the presence of CNP did not affect meiotic arrest (Fig 5A and 5B).

Both gene expression of gap junction protein alpha 1 and 4 (*GJA1*, *GJA4)* in cumulus cells and calcein uptake through gap junctions in COCs derived from small and medium size follicles increased in the presence of CNP. But in the oocyte, *GJA4* gene expression in the presence of TCM or CNP in each group did not show any significant difference [Fig 6, see the experimental design (Fig 1, part II)].

### Developmental competence of COCs in the presence of AREG and PGE2

Optimal concentration of AREG in COCs derived from small size follicles (2 to ≤4 mm) was 300 nM because of the highest CEI (1.93), rate of M II (46.453 ± 0.73), cleavage (75.7± 3.9) and blastocyst rates (41.5± 1.5) (Table 2) (P<0.05).

**Table 2:** Determination of optimal concentration of AREG in COCs derived from small size follicles.

Groups	CEI	M II (%)	Cleavage Rate (%)	Blastocyst Rate (%)
Control	0.21^c^	20.46 ± 2.11 ^b^	63.70 ± 5.03	16.35± 3.07 ^b^
50 nM AREG	0.92 ^b^	42.043 ± 4.83 ^ab^	70.67± 2.22	21.63± 2.70 ^b^
100 nM AREG	1.20^b^	57.143 ± 9.18 ^a^	71.90 ± 3.83	29.06± 4.76 ^ab^
300 nM AREG	1.93 ^a^	46.453 ± 0.73 ^a^	75.71± 3.96	41.52± 1.51 ^a^

CEI: cumulus expansion index, M II (%): Percentage of oocytes at the M II stage. Cleavage and Blastocyst rates were assessed by *in vitro* fertilization in COCs. Different superscripts demonstrate significant differences (P< 0.05) within each column. 5 replicates and the minimum number of oocytes in each replicate were 50, 20 and 20 for assessment of development, MII rate and CEI, respectively.

Optimal concentration of PGE2 in COCs derived from small size follicles (2 to ≤4 mm) was 10 μM because of the highest rate of blastocyst (38.40 ± 0.51), but M II (%) (39.1 ± 1.6) did not show significant difference in comparison with 0.1 μM (36.4 ± 4.1) and 1 μM (31.0 ± 4.0) PGE2 (P>0.05). Also, the cleavage rate was not different between groups. The highest CEI was achieved in the presence of 1 μM PGE2 (P<0.05) (Table 3).

**Table 3:** Determination of optimal concentration of PGE2 in COCs derived from small size follicles.

Groups	CEI	M II (%)	Cleavage Rate (%)	Blastocyst Rate (%)
Control	0.21^c^	21.07 ± 1.02 ^b^	68.79 ± 2.27	20.16 ± 2.94 ^b^
0.1 μM PGE2	1.22 ^b^	31.05 ± 4.07 ^ab^	73.15 ± 2.87	32.53 ± 8.3 ^b^
1 μM PGE2	2.20^a^	36.4 ± 4.1 ^a^	75.18 ± 2.58	30.67 ± 0.21 ^b^
10 μM PGE2	1.42 ^b^	39.18 ± 1.63 ^a^	76.96 ± 3.2	38.40 ± 0.51 ^a^

CEI: cumulus expansion index, M II (%): Percentage of oocytes at the M II stage. Cleavage and Blastocyst rates were assessed by *in vitro* fertilization in COCs. Different superscripts demonstrate significant differences (P< 0.05) within each column. 5 replicates and the minimum number of oocytes in each replicate were 50, 20 and 20 for assessment of development, MII rate, and CEI, respectively.

### Developmental competence of sequential COCs exposure to CNP, AREG and/or PGE2

To achieve this aim, 6 experimental groups were designed [Fig 7, see the experimental design (Fig 1, part III)]. Sequential exposure of COCs to group 3 [TCM+CNP (6 h), then cultured in TCM+AREG (18 h)], group 5 [TCM+CNP (6 h), then cultured in TCM+ AREG+PGE2 (18 h)], and group 6 [TCM+CNP (6 h), then cultured in conventional IVM supplements+AREG+PGE2 (18h)] showed higher blastocyst yield in comparison with TCM (P<0.05). But, group 4 [TCM+CNP (6 h), then cultured in TCM+PGE2 (18 h)] showed lowest blastocyst yield in comparison with the three aforementioned (3, 5, 6) groups (P<0.05) (Fig 7).

Moreover, we checked for any differences between the [TCM+CNP (12 h), then cultured in TCM+AREG+PGE2 (12 h)] and [TCM+CNP (24 h), then cultured in TCM+AREG+PGE2 (24 h)] groups, but the development yields were not different for cleavage (97.56±5, 99±3.24) and blastocyst (26.22±1.5, 23±4.2) rates, respectively.

Also, CEI, cleavage and blastocyst rates of conventional IVM (24 h), [TCM+CNP (6 h), were compared, then cultured in TCM+AREG+PGE2 (18 h)] and [TCM+CNP (6 h), then cultured in conventional IVM supplements + AREG + PGE2 (18 h)]. It was seen that COCs in [TCM+CNP then cultured in conventional IVM supplements + AREG + PGE2] had higher CEI (3.56) and blastocyst rate (41±2.51) compared to conventional IVM (3.1 and 35.9± 5.0) and [TCM+CNP then cultured in TCM+AREG+PGE2] (3 and 34.9 ± 2.1) (P>0.05).

[TCM+CNP (6 h), then cultured in TCM+AREG+PGE2 (18 h)] could only improve the development of oocyte derived from small follicles (P<0.05) [Fig 8, see the experimental design (Fig 1, part III)]. In order to investigate whether [TCM+CNP (6 h), then cultured in TCM+AREG+PGE2 (18 h)] has the same effect on medium size follicles, we compared this treatment with TCM for medium size follicles. The results revealed no significant effect on cleavage and blastocyst rates in medium size follicles.

### Assessment of pluripotency and epigenetic markers in blastocysts derived from small size follicles

The only significant difference in gene expression was for *SOX2* which increased in the [TCM+CNP (6 h), then cultured in TCM+AREG+PGE2 (18 h)] group and for *OCT4 which* decreased in the [TCM*+*CNP (6 h), then cultured in conventional IVM supplements + AREG + PGE2 (18 h)] group (P<0.05). *NANOG*, *CDX2*, and *TET1* did not show any significant differences among the groups (P>0.05) [Fig 9, (see Fig 1, part III)].

## Discussion

One of the limiting factors in the application of IVM is a heterogeneous stage of follicles developing within or between ovaries. The heterogeneous populations of COCs derived from one ovary or different ovaries or from different abattoir have various developmental capacities or outcomes [10, 38]. Differences in morphology, adenosine triphosphate (ATP) content, metabolism, mitochondria distribution, protein, mRNA pattern, and methylation lead to different outcomes in *in vitro* production of the embryo (IVP) [10, 39]. Consistent with previous reports, in the present study, a reduction was observed in the developmental potential of ovine oocytes derived from small (2 to ≤ 4 mm) compared to medium (>4 to 6 mm) follicles, concluding that the follicular size affects developmental competency of COCs.

It is well defined that the isolation of COCs from the natural follicular environment results in spontaneous meiotic progression [40]. Granulosa cells under physiological conditions regulate the expression of transcripts like *NPPC*/*NPR2*, *PTGER2* /*PTGS2*, *AREG/EGFR* involved in oocyte maturation, cumulus expansion, and ovulation through autocrine or paracrine mediators rather than circulating hormones [10, 28]. As we have shown, expression of these transcripts, especially *PTGER2* /*PTGS2*, is lower in CCs isolated from small size follicles compared to medium ones. Also, our data showed that the relative *NPR2* transcript level decreased by passing of time during maturation in CCs derived from small follicles. Thus, the addition of these factors during IVM may improve IVP outcomes.

When CNP binds to guanylyl cyclase (GC)-coupled NPR2, cGMP production increases, and oocytes are maintained at the GV stage [41]. Therefore, it is not surprising to see that the CNP or Npr2 knockout rodent model results in an early resumption of meiosis, concluding the role of CNP in meiotic arrest. Similar functions for NPs were reported in mice, human, goat, cattle, pig, sheep, rat, and cat [42, 43]. In summary, the higher mRNA abundance of NP receptors in the dominant or medium follicles compared to the subordinate or smaller size follicles may be used as an indicator of follicle health and suggests that NPs signaling may regulate steroidogenesis and/or cell proliferation and differentiation [44]. The present study showed that the addition of 1000 nM and 100 nM CNP could arrest the majority of COCs derived from medium and small follicles for 24 h and 12 h, respectively. Recently, some studies have shown 6 h of culture with CNP (100 nM) can enhance oocyte development in cattle and goat [45, 46]. Furthermore, markers of gap junction communication (*GJA4* and *GJA1*) increased in the presence of CNP. Moreover, based on literature, chromatin configuration changes from non-surrounded nucleolus (NSN) to surrounded nucleolus (SN) during oocyte maturation, and this is regulated *in vitro* by NPs [47, 48]. Recently, a novel mechanism for the CNP-induced oocyte meiotic arrest has been introduced in bovine. Based on these results, bovine oocytes have NPR2 receptors and can mediate meiotic arrest [23]. Furthermore, inhibition of meiotic resumption with NPs instead of synthetic reagents like forskolin, PDE inhibitors and specific inhibitors of cyclin-dependent kinases (CDKs) and meiosis promoting factor (MPF) have been shown to have better outcomes as they maintain gap junction activity, and also support key gene expression, which are critical for oocyte development [49–51]. In addition, it has been stated that E2 can mediate NPR2 gene expression and may affect meiotic arrest [22]. Our results showed E2 independently or in the presence of CNP did not affect the meiotic arrest.

On the other hand, the final stage of oocyte maturation and ovulation is mediated by EGF-like peptides after surging with gonadotrophins. The mRNA levels of CNP in granulosa and Npr2 in cumulus cells reduce after LH/hCG treatment despite stimulation of EGF-like factors and activation of EGFR [44, 52]. But, according to our observation and other studies, the level of EGFR transcript decreases in the final stage of *in vitro* maturation in CCs derived from small size follicles. Therefore, it has been stated that the addition of AREG during maturation enhances bovine and porcine oocyte developmental competence [17, 42].

In ovulating follicles, the EGFR signaling cascade involves many other signaling networks in cumulus and granulosa cells, which participate in the development of oocyte competence. Follicle-stimulating hormone (FSH) mediates the induction of *AREG* mRNA via P38 mitogen-activated protein kinases (p38MAPK). AREG also induces *PTGS2* expression via ERK1/2. PGs, also acting via PTGER2 in cumulus cells, provide a secondary, autocrine pathway to regulate expression of *AREG* in COCs. PGE2 acts on a group of G-protein-coupled receptors and can maintain high cAMP levels. PGE2 in our study resulted in mild cumulus expansion, similar to cow [53]. Previous findings indicate that the addition of PGE2 during IVM improves embryonic cell survival of blastocysts and post-hatching development. In this regard, *PTGS2*^-^/^-^ mice present severe failure in the expansion of cumulus cells and extrusion of the first polar body. Our results also indicated that the addition of PGE2 improves cumulus expansion, percentage of MII oocytes, and developmental competency. Besides, we showed that all three factors (AREG, PGE2, and CNP) independently improved the development of COCs derived from small size follicles with TCM as a base medium [8, 14, 17, 43].

We also revealed that sequential exposure of ovine COCs to CNP then to AREG and/or PGE2 improved rate of blastocyst formation in [TCM+CNP, then cultured in TCM+AREG], [TCM+CNP, then cultured in TCM+AREG+PGE2], and [TCM+CNP, then cultured in conventional IVM supplements + AREG + PGE2] compared to TCM, with the best result observed in the latter group. Amongst the treatment groups, the latter also showed higher CEI compared to conventional IVM and [TCM + CNP, then cultured in TCM+AREG+PGE2].

Expansion of the extra-cellular matrix during oocyte maturation is essential for acquiring molecular machinery competence *in vivo* but *in vitro* overexpansion by promoting the hexosamine biosynthesis pathway (HBP) negatively influences *in vitro* oocyte competence [47, 54–56]. Therefore, despite higher CEI in this group, super developmental capacity was not observed.

Expression of embryo quality markers at RNA level revealed that their patterns were not different between conventional IVM, [TCM+CNP, then cultured in TCM+AREG+PGE2], and [TCM+CNP, then cultured in conventional IVM supplements + AREG+PGE2] for *NANOG*, *CDX2*, and *TET1*. But *SOX2* significantly increased in [TCM+CNP (6 h), then cultured in AREG+PGE2], and *OCT4* decreased in [TCM+CNP (6 h), then cultured in conventional IVM supplements + AREG + PGE2]. In mammals, despite specific species differences, the transcription of triad factors (NANOG, *SOX2*, and OCT4) is governed through feedback loops in a steady state. Therefore, knock-down of one factor results in the up-regulation of one or two of the other triad factors [57]. In the studies assessing temporal expression of the triad factors in ruminants, it was shown that the temporal expression of triads is stage-specific dependent, and the expression of *OCT4* is induced during oocyte maturation and declines following development to blastocyst stage, while *SOX2* and *NANOG* are transcribed during maternal/zygote transition and among triads, *SOX2* presents the highest expression relative to references gene [58]. Therefore, the high expression of *SOX2* in the treated group is consistent with the literature, and higher expression of *SOX2* suggests improved zygote genomics activation in the treated group. Further emphasized, improved *in vitro* maturation in this group reflects itself in an improved effect on *SOX2* expression. But, these propositions need further experiments and verifications. One of the shortcomings of this study is that we did not perform concomitant differential staining to observe whether this differential expression may have any effect on total cell numbers and cell allocations.

## Conclusion

Despite interspecies differences among sheep with human and other farm animals, the sequential maturation of sheep oocyte by stimulating NP/NPR2, AREG/EGFR, and PGE2/PTGSR2 system can improve the quality of COCs that have reduced expression of these functional pathways, meaning that COCs derived from small size follicles are less competent due to lower expression of above genes. Therefore, based on the results of this study and others, the supplementation of IVM medium with products of COCs [59] and GCs secreted factors improves developmental capacity and is an alternative to synthetic chemical treatment.

## References

[1] Cha KY, Koo JJ, Ko JJ, Choi DH, Han SY, Yoon TK. Pregnancy after in vitro fertilization of human follicular oocytes collected from nonstimulated cycles, their culture in vitro and their transfer in a donor oocyte program. Fertility and sterility. 1991 1 1;55(1):109–13. 10.1016/s0015-0282(16)54068-0 1986950

[2] Edwards RG. Maturation in vitro of human ovarian oocytes. The Lancet. 1965 11 6;286(7419):926–9. 10.1016/s0140-6736(65)92903-x 4165802

[3] Vuong LN, Ho TM, Gilchrist RB, Smitz J. The Place of In Vitro Maturation in Assisted Reproductive Technology. Fertility & Reproduction. 2019 3 6;1(01):11–5.

[4] Gilchrist RB. Recent insights into oocyte–follicle cell interactions provide opportunities for the development of new approaches to in vitro maturation. Reproduction, Fertility and Development. 2010 12 7;23(1):23–31. 10.1071/RD10225 21366977

[5] Coticchio G, Dal Canto M, Mignini Renzini M, Guglielmo MC, Brambillasca F, Turchi D, et al Oocyte maturation: gamete-somatic cells interactions, meiotic resumption, cytoskeletal dynamics and cytoplasmic reorganization. Human reproduction update. 2015 3 4;21(4):427–54. 10.1093/humupd/dmv011 25744083

[6] Chaube SK. Role of meiotic maturation regulatory factors in developmental competency of mammalian oocytes. HPPI. 2001;24:218–31.

[7] Kwak SS, Yoon JD, Cheong SA, Jeon Y, Lee E, Hyun SH. The new system of shorter porcine oocyte in vitro maturation (18 hours) using≥ 8 mm follicles derived from cumulus-oocyte complexes. Theriogenology. 2014 1 15;81(2):291–301. 10.1016/j.theriogenology.2013.09.028 24220361

[8] Sugimura S, Ritter LJ, Rose RD, Thompson JG, Smitz J, Mottershead DG, et al Promotion of EGF receptor signaling improves the quality of low developmental competence oocytes. Developmental biology. 2015 7 15;403(2):139–49. 10.1016/j.ydbio.2015.05.008 25981108

[9] Yang M, Hall J, Fan Z, Regouski M, Meng Q, Rutigliano HM, et al Oocytes from small and large follicles exhibit similar development competence following goat cloning despite their differences in meiotic and cytoplasmic maturation. Theriogenology. 2016 12 1;86(9):2302–11. 10.1016/j.theriogenology.2016.07.026 27650944

[10] Pandey S, Somal AN, Parmar MS, Gupta S, Chandra V, Sai Kumar G, et al Comparative analysis of developmental and molecular correlates of developmental competence of buffalo oocytes derived from small and large follicles. Indian J Anim Sci. 2017 10 1;87:1194–9.

[11] Celik O, Celik N, Gungor S, Haberal ET, Aydin S. Selective regulation of oocyte meiotic events enhances progress in fertility preservation methods. Biochemistry insights. 2015 1;8:BCI-S28596. 10.4137/BCI.S28596 PMC4577271 26417205

[12] Ouandaogo ZG, Frydman N, Hesters L, Assou S, Haouzi D, Dechaud H, et al Differences in transcriptomic profiles of human cumulus cells isolated from oocytes at GV, MI and MII stages after in vivo and in vitro oocyte maturation. Human Reproduction. 2012 5 22;27(8):2438–47. 10.1093/humrep/des172 22617121

[13] Sugimura S, Kobayashi N, Okae H, Yamanouchi T, Matsuda H, Kojima T, et al Transcriptomic signature of the follicular somatic compartment surrounding an oocyte with high developmental competence. Scientific reports. 2017 7 28;7(1):6815 10.1038/s41598-017-07039-5 28755009 PMC5533789

[14] Zhang T, Fan X, Li R, Zhang C, Zhang J. Effects of pre-incubation with C-type natriuretic peptide on nuclear maturation, mitochondrial behavior, and developmental competence of sheep oocytes. Biochemical and biophysical research communications. 2018 2 26;497(1):200–6. 10.1016/j.bbrc.2018.02.054 29428720

[15] Zhang Y, Yan Z, Qing Q, Nisenblat V, Yu Y, Wang T, et al Transcriptome Landscape of Human Oocytes and Granulosa Cells Throughout Folliculogenesis. bioRxiv. 2018 1 1:285445.

[16] Liu C, Sun W, Zhuo G, Zhang Z. Impacts of macrophage colony-stimulating factor (M-CSF) on the expression of natriuretic peptide precursor type C (NPPC) and regulation of meiotic resumption. Gynecological Endocrinology. 2019 4 3;35(4):320–3. 10.1080/09513590.2018.1532989 30767584

[17] Siqueira LC, Barreta MH, Gasperin B, Bohrer R, Santos JT, Junior JB, et al Angiotensin II, progesterone, and prostaglandins are sequential steps in the pathway to bovine oocyte nuclear maturation. Theriogenology. 2012 6 1;77(9):1779–87. 10.1016/j.theriogenology.2011.12.022 22365701

[18] Sugimura S, Ritter LJ, Sutton-McDowall ML, Mottershead DG, Thompson JG, Gilchrist RB. Amphiregulin co-operates with bone morphogenetic protein 15 to increase bovine oocyte developmental competence: effects on gap junction-mediated metabolite supply. Molecular human reproduction. 2014 2 19;20(6):499–513. 10.1093/molehr/gau013 24557840

[19] Fang Y, Zhang X, Zhang J, Zhong R, Zhou D. Global DNA methylation and related mRNA profiles in sheep oocytes and early embryos derived from pre-pubertal and adult donors. Animal reproduction science. 2016 1 1;164:144–51. 10.1016/j.anireprosci.2015.11.022 26686460

[20] Brown HM, Dunning KR, Sutton-McDowall M, Gilchrist RB, Thompson JG, Russell DL. Failure to launch: aberrant cumulus gene expression during oocyte in vitro maturation. Reproduction. 2017 3 1;153(3):R109–20. 10.1530/REP-16-0426 27879344

[21] De Cesaro MP, Dos Santos JT, Ferst JG, Nobrega JE Jr, Rosa PR, Rovani MT, et al Natriuretic peptide system regulation in granulosa cells during follicle deviation and ovulation in cattle. Reproduction in domestic animals. 2018 6;53(3):710–7. 10.1111/rda.13161 29537121

[22] Franciosi F, Coticchio G, Lodde V, Tessaro I, Modina SC, Fadini R, et al Natriuretic peptide precursor C delays meiotic resumption and sustains gap junction-mediated communication in bovine cumulus-enclosed oocytes. Biology of reproduction. 2014 9 1;91(3):61–1. 10.1095/biolreprod.114.118869 25078681

[23] Kim SM, Kim SH, Cho KW, Young S. Expression of C-type Natriuretic Peptide and its Specific Guanylyl Cyclase-Coupled Receptor in Pig Ovarian Granulosa Cells.

[24] Xi G, An L, Jia Z, Tan K, Zhang J, Wang Z, et al Natriuretic peptide receptor 2 (NPR2) localized in bovine oocyte underlies a unique mechanism for C-type natriuretic peptide (CNP)-induced meiotic arrest. Theriogenology. 2018 1 15;106:198–209. 10.1016/j.theriogenology.2017.09.003 29080478

[25] Shimada M, Umehara T, Hoshino Y. Roles of epidermal growth factor (EGF)-like factor in the ovulation process. Reproductive medicine and biology. 2016 10 1;15(4):201–16. 10.1007/s12522-016-0236-x 29259438 PMC5715866

[26] Nuttinck F, Gall L, Ruffini S, Laffont L, Clement L, Reinaud P, et al PTGS2-related PGE2 affects oocyte MAPK phosphorylation and meiosis progression in cattle: late effects on early embryonic development. Biology of reproduction. 2011 6 1;84(6):1248–57. 10.1095/biolreprod.110.088211 21293029

[27] Marei WF, Abayasekara DR, Wathes DC, Fouladi-Nashta AA. Role of PTGS2-generated PGE2 during gonadotrophin-induced bovine oocyte maturation and cumulus cell expansion. Reproductive biomedicine online. 2014 3 1;28(3):388–400. 10.1016/j.rbmo.2013.11.005 24447957

[28] Blaha M, Prochazka R, Adamkova K, Nevoral J, Nemcova L. Prostaglandin E2 stimulates the expression of cumulus expansion-related genes in pigs: the role of protein kinase B. Prostaglandins & other lipid mediators. 2017 5 1;130:38–46. 28389370 10.1016/j.prostaglandins.2017.04.001

[29] Takahashi T, Hagiwara A, Ogiwara K. Prostaglandins in teleost ovulation: A review of the roles with a view to comparison with prostaglandins in mammalian ovulation. Molecular and cellular endocrinology. 2018 2 5;461:236–47. 10.1016/j.mce.2017.09.019 28919301

[30] Jahromi BN, Mosallanezhad Z, Matloob N, Davari M, Ghobadifar MA. The potential role of granulosa cells in the maturation rate of immature human oocytes and embryo development: A co-culture study. Clinical and experimental reproductive medicine. 2015 9 1;42(3):111–7. 10.5653/cerm.2015.42.3.111 26473111 PMC4604294

[31] Sahu K, Gupta A, Sharma A, Tiwari M, Pandey AN, Prasad S, et al Role of granulosa cell mitogen-activated protein kinase 3/1 in gonadotropin-mediated meiotic resumption from diplotene arrest of mammalian oocytes. Growth Factors. 2018 3 4;36(1–2):41–7. 10.1080/08977194.2018.1475372 29842809

[32] Pezhman M, Hosseini SM, Ostadhosseini S, Varnosfaderani SR, Sefid F, Nasr-Esfahani MH. Cathepsin B inhibitor improves developmental competency and cryo-tolerance of in vitro ovine embryos. BMC developmental biology. 2017 12;17(1):10 10.1186/s12861-017-0152-2 28676034 PMC5496377

[33] Sefid F, Ostadhosseini S, Hosseini SM, Zadegan FG, Pezhman M, Esfahani MH. Vitamin K2 improves developmental competency and cryo-tolerance of in vitro derived ovine blastocyst. Cryobiology. 2017 8 1;77:34–40. 10.1016/j.cryobiol.2017.06.001 28596081

[34] Varnosfaderani SR, Ostadhosseini S, Hajian M, Hosseini SM, Khashouei EA, Abbasi H, et al Importance of the GDF9 signaling pathway on cumulus cell expansion and oocyte competency in sheep. Theriogenology. 2013 9 15;80(5):470–8. 10.1016/j.theriogenology.2013.05.009 23764009

[35] Varnosfaderani SR, Ostadhosseini S, Hosseini SM, Hajian M, Nasr-Esfahani MH. In vitro development of ovine oocytes cultured in the presence of GDF9 at chemically defined or undefined maturation medium. Small Ruminant Research. 2014 7 1;120(1):78–83.

[36] Azari-Dolatabad N, Rahmani HR, Hajian M, Ostadhosseini S, Hosseini SM, Nasr-Esfahani MH. Effects of cilostamide and/or forskolin on the meiotic resumption and development competence of growing ovine oocytes selected by brilliant cresyl blue staining. Theriogenology. 2016 5 1;85(8):1483–90. 10.1016/j.theriogenology.2016.01.008 26879998

[37] Vanderhyden BC, Caron PJ, Buccione R, Eppig JJ. Developmental pattern of the secretion of cumulus expansion-enabling factor by mouse oocytes and the role of oocytes in promoting granulosa cell differentiation. Developmental biology. 1990 8 1;140(2):307–17. 10.1016/0012-1606(90)90081-s 2115479

[38] Moradi M, Riasi A, Ostadhosseini S, Hajian M, Hosseini M, Hosseinnia P, et al Expression profile of FGF receptors in preimplantation ovine embryos and the effect of FGF2 and PD173074. Growth Factors. 2015 9 3;33(5–6):393–400. 10.3109/08977194.2015.1102138 26768755

[39] Palmerini MG, Nottola SA, Leoni GG, Succu S, Borshi X, Berlinguer F, et al In vitro maturation is slowed in prepubertal lamb oocytes: ultrastructural evidences. Reproductive Biology and Endocrinology. 2014 12;12(1):115. 25421574 10.1186/1477-7827-12-115 PMC4247763

[40] Leoni GG, Palmerini MG, Satta V, Succu S, Pasciu V, Zinellu A, et al Differences in the kinetic of the first meiotic division and in active mitochondrial distribution between prepubertal and adult oocytes mirror differences in their developmental competence in a sheep model. PLoS One. 2015 4 20;10(4):e0124911 10.1371/journal.pone.0124911 25893245 PMC4403920

[41] Oskouei BS, Pashaiasl M, Heidari MH, Salehi M, Veladi H, Pakdel FG, et al Evaluation of mouse oocyte in vitro maturation developmental competency in dynamic culture systems by design and construction of a lab on a chip device and its comparison with conventional culture system. Cell Journal (Yakhteh). 2016 7;18(2):205. 10.22074/cellj.2016.4315 PMC4988419 27540525

[42] Conti M, Andersen CB, Richard F, Mehats C, Chun SY, Horner K, et al Role of cyclic nucleotide signaling in oocyte maturation. Molecular and cellular endocrinology. 2002 2 22;187(1–2):153–9. 10.1016/s0303-7207(01)00686-4 11988323

[43] Romero S, Sánchez F, Lolicato F, Van Ranst H, Smitz J. Immature oocytes from unprimed juvenile mice become a valuable source for embryo production when using C-type natriuretic peptide as essential component of culture medium. Biology of reproduction. 2016 9 1;95(3):64–1. 10.1095/biolreprod.116.139808 27488026

[44] Sánchez F, Lolicato F, Romero S, De Vos M, Van Ranst H, Verheyen G, et al An improved IVM method for cumulus-oocyte complexes from small follicles in polycystic ovary syndrome patients enhances oocyte competence and embryo yield. Human reproduction. 2017 8 30;32(10):2056–68. 10.1093/humrep/dex262 28938744

[45] dos Santos JT, De Cesaro MP, Ferst JG, Dau AM, da Rosa PR, Pasqual BM, et al Luteinizing hormone upregulates NPPC and downregulates NPR3 mRNA abundance in bovine granulosa cells through activation of the EGF receptor. Theriogenology. 2018 10 1;119:28–34. 10.1016/j.theriogenology.2018.06.012 29960164

[46] Soto-Heras S, Paramio MT, Thompson JG. Effect of pre-maturation with C-type natriuretic peptide and 3-isobutyl-1-methylxanthine on cumulus-oocyte communication and oocyte developmental competence in cattle. Animal reproduction science. 2019 3 1;202:49–57. 10.1016/j.anireprosci.2019.01.007 30772104

[47] Soto-Heras S, Menéndez-Blanco I, Catalá MG, Izquierdo D, Thompson JG, Paramio MT. Biphasic in vitro maturation with C-type natriuretic peptide enhances the developmental competence of juvenile-goat oocytes. PloS one. 2019 8 23;14(8):e0221663 10.1371/journal.pone.0221663 31442286 PMC6707569

[48] Zuccotti M, Merico V, Cecconi S, Redi CA, Garagna S. What does it take to make a developmentally competent mammalian egg?. Human reproduction update. 2011 3 28;17(4):525–40. 10.1093/humupd/dmr009 21444328

[49] Sun MJ, Zhu S, Li YW, Lin J, Gong S, Jiao GZ, et al An essential role for the intra-oocyte MAPK activity in the NSN-to-SN transition of germinal vesicle chromatin configuration in porcine oocytes. Scientific reports. 2016 3 24;6:23555 10.1038/srep23555 27009903 PMC4806380

[50] Kawashima I, Okazaki T, Noma N, Nishibori M, Yamashita Y, Shimada M. Sequential exposure of porcine cumulus cells to FSH and/or LH is critical for appropriate expression of steroidogenic and ovulation-related genes that impact oocyte maturation in vivo and in vitro. Reproduction. 2008 7 1;136(1):9 10.1530/REP-08-0074 18456902

[51] Caixeta FM, Sousa RV, Guimarães AL, Leme LO, Sprícigo JF, Netto SS, et al Meiotic arrest as an alternative to increase the production of bovine embryos by somatic cell nuclear transfer. Zygote. 2017 2;25(1):32–40. 10.1017/S0967199416000289 27780485

[52] Egbert JR, Yee SP, Jaffe LA. Luteinizing hormone signaling phosphorylates and activates the cyclic GMP phosphodiesterase PDE5 in mouse ovarian follicles, contributing an additional component to the hormonally induced decrease in cyclic GMP that reinitiates meiosis. Developmental biology. 2018 3 1;435(1):6–14. 10.1016/j.ydbio.2018.01.008 29341896 PMC5818284

[53] Shimada M, Hernandez-Gonzalez I, Gonzalez-Robayna I, Richards JS. Paracrine and autocrine regulation of epidermal growth factor-like factors in cumulus oocyte complexes (COCs) and granulosa cells: key roles for prostaglandin synthase 2 and progesterone receptor. Molecular Endocrinology. 2006 6 1;20(6):1352–65. 10.1210/me.2005-0504 16543407

[54] Nuttinck F, Jouneau A, Charpigny G, Hue I, Richard C, Adenot P, et al Prosurvival effect of cumulus prostaglandin G/H synthase 2/prostaglandin2 signaling on bovine blastocyst: impact on in vivo posthatching development. Biology of reproduction. 2017 3 1;96(3):531–41. 10.1095/biolreprod.116.145367 28339853 PMC5819843

[55] Thompson JG, Lane M, Gilchrist RB. Metabolism of the bovine cumulus-oocyte complex and influence on subsequent developmental competence. Society of Reproduction and Fertility supplement. 2007 4 1;64:179 10.5661/rdr-vi-179 17491147

[56] Hu S, Rao M, Lei H, Wu Y, Wang Y, Ke D, et al Expression patterns of p38αMAPK during follicular development in the ovaries of neonatal rats. Acta histochemica. 2017 6 1;119(5):538–42. 10.1016/j.acthis.2017.05.007 28606727

[57] Habibi R, Hosseini SM, Zadegan FG, Hajian M, Ostadhosseini S, Vash NT, et al Functional characterization of NANOG in goat pre-implantation embryonic development. Theriogenology. 2018 10 15;120:33–9. 10.1016/j.theriogenology.2018.07.023 30092372

[58] HosseinNia P, Hajian M, Tahmoorespur M, Hosseini SM, Ostadhosseini S, et al Expression Profile of Developmentally Important Genes in preand peri-Implantation Goat Embryos Produced In Vitro. International journal of fertility & sterility. 2016 10;10(3):310. 27695614 10.22074/ijfs.2016.4659 PMC5023042

[59] Mottershead DG, Sugimura S, Al-Musawi SL, Li JJ, Richani D, White MA, et al Cumulin, an oocyte-secreted heterodimer of the transforming growth factor-β family, is a potent activator of granulosa cells and improves oocyte quality. Journal of Biological Chemistry. 2015 9 25;290(39):24007–20. 10.1074/jbc.M115.671487 26254468 PMC4583026

