# Supplementary material for: Granulosa secreted factors improve the developmental competence of cumulus oocyte complexes from small antral follicles in sheep
Source: PLoS One. 2020 Mar 17;15(3):e0229043. doi: 10.1371/journal.pone.0229043 (PMC7077809; doi:10.1371/journal.pone.0229043)
Supplement: Attachment — Submitted filename: reply to PLOS ONE .docx [file pone.0229043.s002.docx]

Dear Dr. Wilfried A. Kues,

Hereby, we are submitting the revised version of our manuscript entitled: “Granulosa secreted factors improve the developmental competence of cumulus oocyte complexes from small antral follicles” (PONE-D-19-25159R2).

We would like to thank you and respected reviewer for valuable comments. The original comment is in black and our response is in blue. For better check out, the implemented changes were defined as underlined in the revised manuscript. We hope it has now acquired the high status to meet the requirements for publication in your esteemed journal.

Best Regards

M. H. Nasr-Esfahani,

Email: mh.nasr-esfahani@royaninstitute.org

**Reviewer comment:**

Reviewer #1: The revised manuscript has been significantly improved and all the major concerns were addressed. Compared to the first version, major improvements have been made to the grammar making the manuscript much easier to read and understand. However, some minor grammar mistakes still persist.

Reply: The authors appreciate the reviewer’s valuable comment. All grammar mistakes throughout the manuscript were carefully edited, and we hope the changes are satisfactory. All changes were underlined in the text.
